# The expression of beclin-1, an autophagic gene, in hepatocellular carcinoma associated with clinical pathological and prognostic significance

**DOI:** 10.1186/1471-2407-14-327

**Published:** 2014-05-09

**Authors:** Dong-Mei Qiu, Gui-Lan Wang, Li Chen, Yu-Yin Xu, Song He, Xiao-Lei Cao, Jing Qin, Jia-Ming Zhou, Yi-Xin Zhang, Qun E

**Affiliations:** 1 Department of Pathological Anatomy, Nantong University, Qixiu road 19#, Nantong, Jiangsu Province, China 226001; 2 Nantong Tumor Hospital, Nantong, P.R. China

**Keywords:** Beclin-1, Autophagy, HCC, Prognosis proliferation, Apoptosis, Microvessels

## Abstract

**Background:**

A role for autophagy, a conserved cellular response to stress, has recently been demonstrated in human cancers. Aberrant expression of Beclin-1, an important autophagic gene, has been reported in various human cancers. In the present study, we investigated the significance and relationship between Beclin-1 expression and cell proliferation, apoptosis, microvessel density (MVD) and clinical pathological changes or prognosis in human hepatocellular carcinoma (HCC).

**Methods:**

A total of 103 primary HCC patients were involved in the study. Expression of Beclin-1, PCNA, NET-1, Bcl-2, Bax, Survivin in cancer cells and CD34 in stromal microvessels were evaluated immunohistochemically in tissue microarrays comprising 103 cases of HCC and 57 matched adjacent nontumor liver tissues. Correlations between clinicopathological characteristics and survival of HCC patients were explored.

**Results:**

The positive rate of Beclin-1 was significantly lower in HCC tissues than adjacent tissues (72.8 vs. 89.5%, χ2 = 6.085, *P* = 0.015). In HCC, Beclin-1 expression was negatively correlated with cirrhosis background (r = −0.216, *P* = 0.029), Edmondson grade (r = −0.249, *P* = 0.011), vascular invasion (r = −0.246, *P* = 0.012), PCNA (r = −0.242, *P* = 0.014), NET-1 (r = −0.245, *P* = 0.013), anti-apoptosis protein Bcl-2 (r = −0.245, *P* = 0.013) and MVD (r = −0.292, *P* = 0.003), and positively correlated with pro-apoptosis protein Bax (r = 0.242, *P* = 0.014).

Significant differences in the 5-year survival rates were seen among patients with Beclin-1 strong positive (++) (59.1%, 13/22), moderate positive (+) (28.3%, 15/53) and weak negative expression (−) (14.6%, 7/28) (*P* = 0.043). Significant differences were detected between Beclin-1 (++) and either Beclin-1 (+) (*P* = 0.036) or Beclin-1 (−) groups (*P* = 0.008), but no significant difference between Beclin-1 (+) and Beclin-1 (−) groups (*P* = 0.281) was observed.

Survival rates were positively related to high Beclin-1 co-expressed with low PCNA, NET-1, or Bcl-2, lower MVD, and high Bax. Univariate and multivariate Cox regression analysis revealed that Beclin-1 expression was an independent indicator for overall survival in HCC patients (*P* < 0.05).

**Conclusions:**

The pathogenesis and progression of HCC are associated with reduced autophagy. The expression of Beclin-1 and Bax in HCC tissues may provide a synergistic effect towards inhibiting HCC proliferation, infiltration, metastasis and angiogenesis. Beclin-1 expression may be a valuable prognostic marker of HCC.

## Background

Liver cancer is the sixth most common cancer and the third leading cause of cancer death worldwide. A total of 749,000 new cases of liver cancer worldwide were estimated in 2008, causing more than 696,000 deaths [1]. Hepatocellular carcinoma (HCC) is the most common type of liver cancer and one of the most prevalent malignant tumors worldwide [2]. Currently, hepatic resection is the most common treatment modality for HCC and one of the most effective interventions for achieving long-term survival [3,4]. Most HCC patients are diagnosed at an advanced stage with underlying liver dysfunction [5], and have very poor prognoses owing to the low survival. Although several molecular factors and histological features have been reported to be associated with the prognosis of HCC [6,7] more effective biomarkers are necessary to predict the clinical outcome of patients with HCC.

Autophagy is a genetically programmed process that enables the recycling of long-lived proteins or damaged organelles [8,9]. Several studies have demonstrated that autophagy plays an important role in the development and progression of cancer [10,11]. However, the role of autophagy in the growth and metastasis of human carcinomas is not completely understood. Therefore, autophagy has become a critical focus in cancer research [12-14]. Loss of autophagy is likely to contribute to tumor progression by promoting genome damage and instability. Approximately 30 specific genes that regulate autophagy have been identified in yeast, with 16 homologues in human [15]. Among these genes, Beclin-1 plays a key role in mammalian autophagy [16]. Beclin-1, the mammalian counterpart of the yeast Atg6 gene, is part of a type III phosphatidylinositol 3-kinase complex required for autophagic vesicle formation [17-20]. Abnormal expression of Beclin-1 has been found in human melanoma [21], colon [22], ovarian [23] and brain cancers [24]. The aim of this study was to investigate the expression of Beclin-1 in HCC tissues, and its relation to clinicopathological features and prognostic significance.

## Methods

### Patients and follow-up

This was a retrospective study based on archived materials. The study group comprised 103 HCC patients who were diagnosed with HCC and had undergone curative resection in Nantong Cancer Hospital and the Affiliated Hospital of Nantong University between January 2003 and December 2004. The patients were selected according to the following criteria: (a) primary HCC, and (b) previously untreated and with surgery as the first treatment. Therefore, analysis of the data in this series would reflect actual impact of the tumor biology on the clinical outcome.

All patients had regular follow-up. The follow-up period was defined as the duration from the date of operation to the date of either death or the last follow-up. The follow-up period ranged from 13 to 72 months with a median of 58.4 months. The last follow-up was January 2010. Deaths from other causes were treated as censored cases. Overall survival (OS) was evaluated by the duration between the dates of surgery and death.

Among the 103 patients, 85 were male and 18 were female, and the ratio of male to female was 4.8:1 (82.5 and 17.5%). The ages of the patients ranged from 21 to 79 years old with a median of 49 years old. All patients were diagnosed and histopathologically confirmed with HCC, and had complete clinical and pathological records including medical records, chest roentgenograms, whole body computed tomography films, bone and brain scanning data. The surgery records were reviewed and the confirmed pathological diagnosis, tumor size, related hepatitis/liver cirrhosis, metastasis, and serum alpha fetoprotein (AFP) values and other relevant data were analyzed. Negative controls were established from 57 matched adjacent non-tumor liver tissues that were derived from 113 cases of HCC.

The study was approved by the Ethics Committee of the Nantong Cancer Hospital, and all the patients signed informed consent. To determine factors affecting survival after operation, conventional variables together with Beclin-1 expression were determined in the 103 patients.

### Tissue microarrays

Tissue microarrays (TMA) were constructed according to the method of E. Qun (Patent Number: ZL 2008 1 0022 170.4). Briefly, all HCC tissues were stained by H&E and reviewed by two histopathologists. Representative areas free from necrotic and hemorrhagic materials were marked in paraffin blocks. Two cylindrical tissue cores (1.6 mm diameter) were removed from the donor blocks and transferred to the recipient paraffin blocks, and their planar array positions were noted. Three different TMA blocks were constructed. Each contained over 100 cylinders and the final TMAs consisted of 103 cases of HCC and 57 cases of adjacent non-tumor tissues (ANT). Consecutive sections (4-μm thick) were cut from the array blocks and placed on adhesion microscope slides for immunohistochemical staining.

### Immunohistochemistry staining

The Envision+/DAB analysis method was performed on formalin-fixed, paraffin-embedded 5-μm sections from all patients for the detection of Beclin-1, PCNA, NET-1, Bcl-2, Bax and Survivin in cancer cells and CD34 in stromal microvessels. Ten consecutive TMA sections were prepared from each TMA block and stained. The paraffin slides were dewaxed in xylene and microwaved. For antigen retrieval, slides were heated at 95°C for 10 min in sodium citrate buffer (10 mM sodium citrate monohydrate, pH 6.0). The slides were allowed to cool for 20 min at room temperature and then incubated in Envision + peroxidase blocking solution (Dakocytomation, Glostrup, Denmark) for 5 min and rinsed with 0.05% Tris-buffered saline (TBS)/Tween 20 buffer, pH 7.4. The slides were then incubated with primary antibodies for 30 min at room temperature. Anti-Beclin-1 monoclonal antibodies (diluted 1:100) were obtained from ProSci Inc. Mouse monoclonal antibodies against human PCNA, NET-1, Bcl-2, Bax, Survivin and CD34 were purchased from Zymed Laboratories (South San Francisco, California, USA). The slides were washed with 0.05% Tween 20 in TBS (pH 7.4). Detection was achieved with the DAKO Envision+/HRP system (DAKO, Carpinteria, CA, USA). The color was developed by a 15 min incubation with a diaminobenzidine (DAB) solution (DAB kit IL1-9032) (Fuzhou Maixin Biotech. Co., Ltd., China), and sections were slightly counterstained with hematoxylin. Positive controls (follicular B cell lymphoma tissues with strong nuclear PCNA and cytoplasmic Bcl-2, hepatocellular carcinoma with cytoplasmic Beclin-1, NET-1, Bax and Survivin) and negative controls (TBS was substituted for primary antibody at the same concentration) were performed for each immunohistochemical run.

### Staining patterns and evaluation

Stained sections were evaluated in a blinded manner by two independent investigators without prior knowledge of the clinical information using an immunoreactive score (IRS). Scores were assigned for the intensity and percentage of positive staining of the cytoplasm/nucleus in the whole cylinder. Briefly, the IRS assigns subscores for immunoreactive distribution (0–4) and intensity (0–3), and multiplies the subscores to yield the IRS score. The percent positivity was scored as “0” (<5%), “1” (5–25%), “2” (>25–50%), “3” (>50–75%) or “4” (>75%). The staining intensity was scored as “0” (no staining), “1” (weakly stained), “2” (moderately stained) or “3” (heavily stained). In cases where differences between duplicate tissue cores were observed, the higher score was considered to be the final score. The final expression scores of Beclin-1, PCNA, NET-1, Bcl-2, Bax and Survivin were calculated with the value of percent positivity score multiplied by staining intensity score, which ranged from 0 to 12. Intratumorally, the protein expression was defined as follows: negative (−) (score 0), low expression (+) (score 1–6) and high expression (++) (score >6).

CD34 was mainly expressed in endothelial cells of microvessels with a scattered pattern of expression. The densest staining zones were selected at the invading tumor front by an image-analyzed system. The final microvessel score was the mean of the vessel counting number obtained from these fields. Only blood vessels with a clearly defined lumen or a linear vessel shape, but not single endothelial cells, were taken into account. For MVD, a cutoff point of 15 under high power view (×200) was used to define the overexpression group versus the low expression group.

### Statistical analysis

Differences between cancer tissues and adjacent non-tumor tissues were tested for significance using Wilcoxon signed rank tests. The Spearman rank correlation test was performed to determine the association of Beclin-1 expression with either cell proliferation markers (PCNA and NET-1), apoptosis markers (Bcl-2, Bax and Survivin), MVD, or clinicopathological characteristics (pathologic classification, TNM stage, tumor size). Univariate survival analyses were used to examine prognostic significance of Beclin-1 expression. Curves for overall survival (OS) were drawn according to the Kaplan–Meier method and difference was analyzed by applying the log-rank test for univariate survival analysis. In accordance with results from Cox univariate regression analyses, significant factors were evaluated by multivariate regression analyses to determine independent prognostic factors. SPSS 17.0 software (SPSS Inc., Chicago, IL, USA) was used for all statistical analyses and a *P* value <0.05 was considered significant.

## Results

### Beclin-1 expression in HCC and ANT tissues

**Figure 1:**
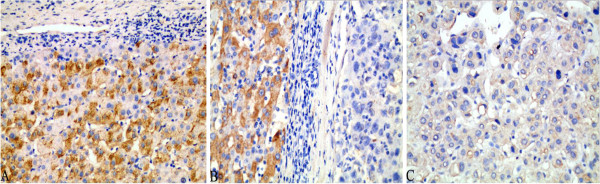
**Beclin-1 exhibited cytoplasmic staining in adjacent non-tumor (ANT) tissues (A), bordeling site between HCC and ANT (B: left, ANT; right: HCC), and HCC (C), respectively.** Beclin-1 expression was stronger in ANT than in HCC. Magnification × 400.

The expression of Beclin-1 in 103 cases of HCC tissues and 57 cases of ANT was examined by immunohistochemical analysis (Figure 1, Table 1). A significant difference was observed in Beclin-1 expression between HCC and ANT samples (χ2 = 6.085, *P* = 0.015).

**Table 1:** Beclin-1 expression in HCC and ANT tissues

**Sample**	**Cases**	**Beclin-1 expression**			**χ**^**2**^**/P**
		**(−) %**	**(+) %**	**(++) %**	
HCC	103	28 27.4	53 51.4	22 21.4	χ2=6.085
ANT	57	6 10.5	31 54.4	20 35.1	p=0.015

### Association of Beclin-1 expression with clinicopathologic features of HCC

**Figure 2:**
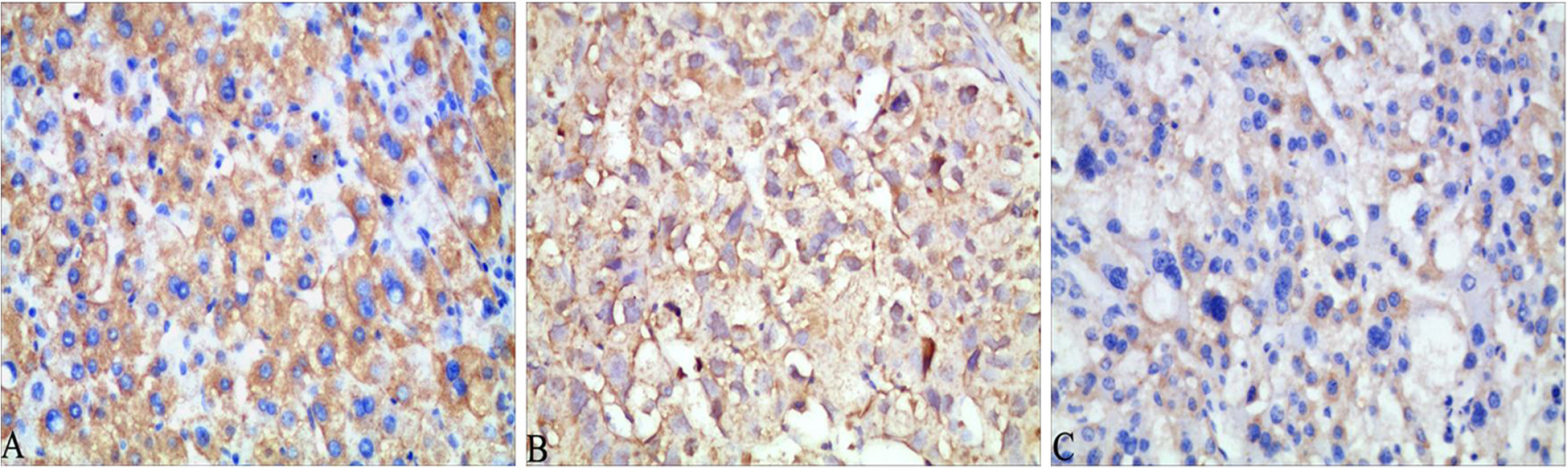
**Beclin-1 expression in well-differentiated HCC with Edmondson I (A); moderately differentiated HCC with Edmondson II grade (B); and poorly differentiated HCC with III–IV grade (C).** Beclin-1 was expressed in the cytoplasm of HCC cells, and with the increased grade of HCC, Beclin-1 expression was lower. Magnification × 400.

**Figure 3:**
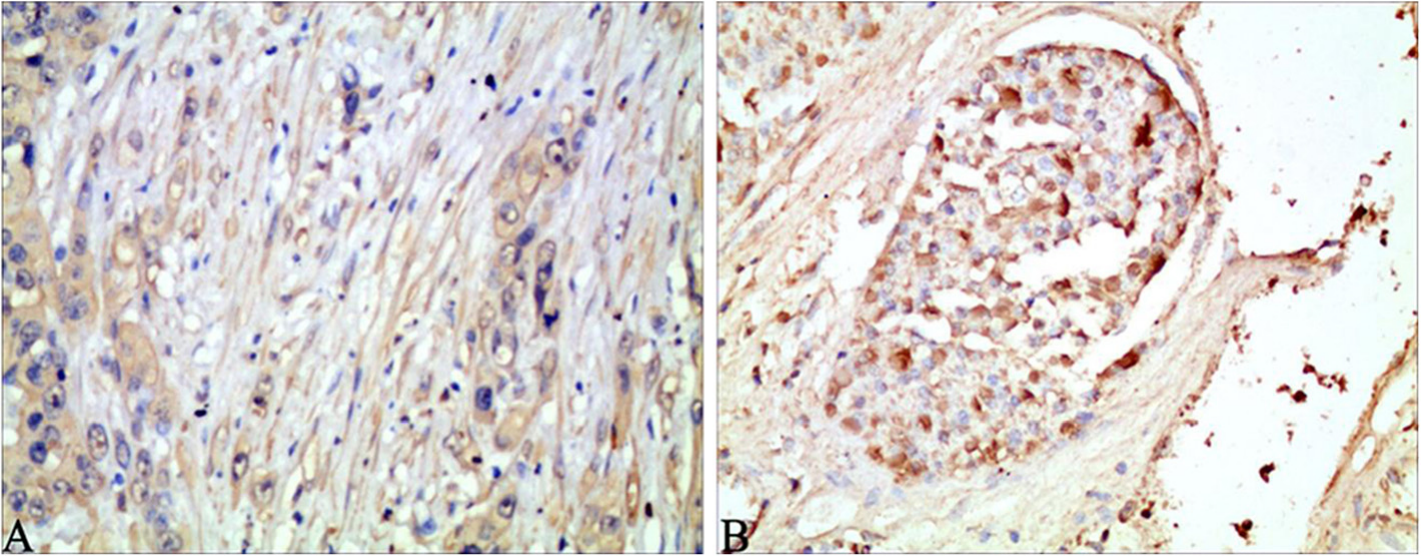
**Beclin-1 expression in HCC with liver cirrhosis (A) and vascular invasion (B).** Low positive signal of Beclin-1 staining indicated its low expression level. Magnification × 200.

The correlation of Beclin-1 expression with the clinicopathologic characteristics of HCC is shown in Table 2. Intratumoral Beclin-1 expression was negatively correlated with HCC Edmondson grade (Figure 2A–C) (*P* = 0.011). Beclin-1 expression was stronger in HCC with Edmondson I–II grade (83.7%, 41/49) than HCC with III–IV grade (63.0%, 34/54). Similarly, the positive rate of Beclin-1 expression in HCC with cirrhosis was 69.9% (58/83) and without cirrhosis was 85.0% (17/20). Beclin-1 expression also negatively correlated with HCC with cirrhosis background (*P* = 0.029) (Figure 3A). The positive rate of Beclin-1 expression in HCC with vascular invasion was 32.0% (18/33) and expression in HCC without vascular invasion was 68.0% (57/70). Beclin-1 expression also negatively correlated with HCC with vascular invasion (*P* = 0.012) (Figure 3B). There were no significant associations between Beclin-1 expression and other clinicopathologic features.

**Table 2:** Correlation between Beclin-1 expression and clinicopathologic characteristics in HCC

**Characteristics**	**N**	**Beclin-1 expression**						**R (X**^**2**^**# )/P**
		**(−)**	**%**	**(+)**	**%**	**(++)**	**%**	
Age								1.382#/0.155
<50 y	53	12	22.6	28	52.8	13	24.5	
≥50 y	50	16	32	25	50	9	18	
Sex								4.360#/0.175
Male	85	23	27.1	47	55.3	15	17.6	
Female	18	5	27.8	6	33.3	7	38.9	
AFP								−0.096/0.334
<50 ng/ml	39	8	20.5	22	56.4	9	23.1	
≥50 ng/ml	64	20	31.3	31	48.4	13	20.3	
HbsAg								−0.111/0.264
Negative	20	4	20	10	50	6	30	
Positive	83	24	28.9	43	51.8	16	19.3	
Liver cirrhosis								0.216 /0.029*
No	20	3	15	9	45	8	40	
Yes	83	25	30.1	44	53	14	16.9	
Tumor size								−0.035/0.726
≤2 cm	14	3	21.4	7	50.0	4	28.6	
2 cm-10 cm	78	22	28.2	41	52.6	15	19.2	
>10 cm	11	3	27.3	5	45.5	3	27.3	
Edmondson grades								−0.249/0.011*
I-II	49	8	16.3	27	55.1	14	28.6	
III-IV	54	20	37	26	48.1	8	14.8	
TNM stage								0.146/0.142
I	10	2	10	4	40	4	50	
II	41	7	17.1	27	71.1	7	18.4	
III	36	13	30.7	16	46.2	7	23.1	
IV	16	6	25	6	37.5	4	37.5	
Capsule integrity								−0.054/0.588
Yes	67	16	23.9	34	50.7	17	25.4	
No	36	12	33.3	19	52.8	5	13.9	
Vascular invasion								−0.246/0.012*
No	70	13	18.6	40	57.1	17	24.3	
Yes	33	15	45.5	13	39.4	5	15.2	
Tumor number								−0.158/0.111
Single	73	17	23.6	38	52.05	18	25	
Multiple	30	11	37.9	15	50.0	4	13.8	

*, p<0.05.

### The association of Beclin-1 expression with proliferation and apoptosis-related proteins and MVD in HCC

**Figure 4:**
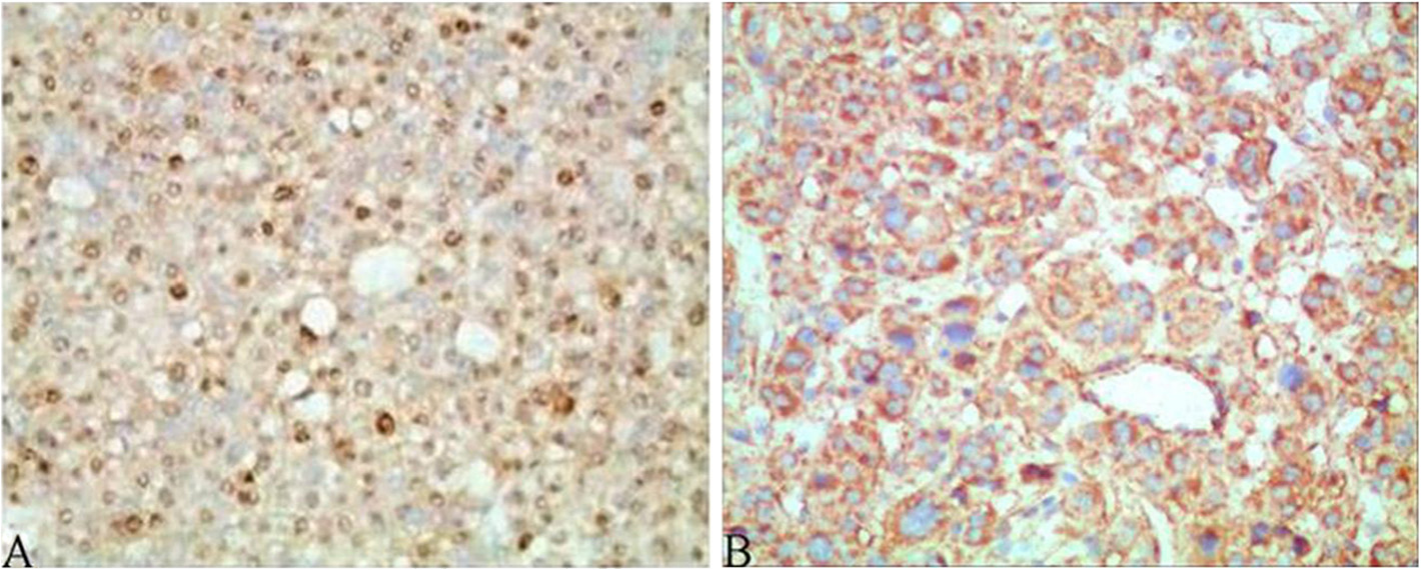
**The expression of PCNA (A) and NET-1 (B) in HCC.** PCNA strong positive staining was seen in the nucleus and NET-1 was detected in the cytoplasm. Magnification × 400.

**Figure 5:**
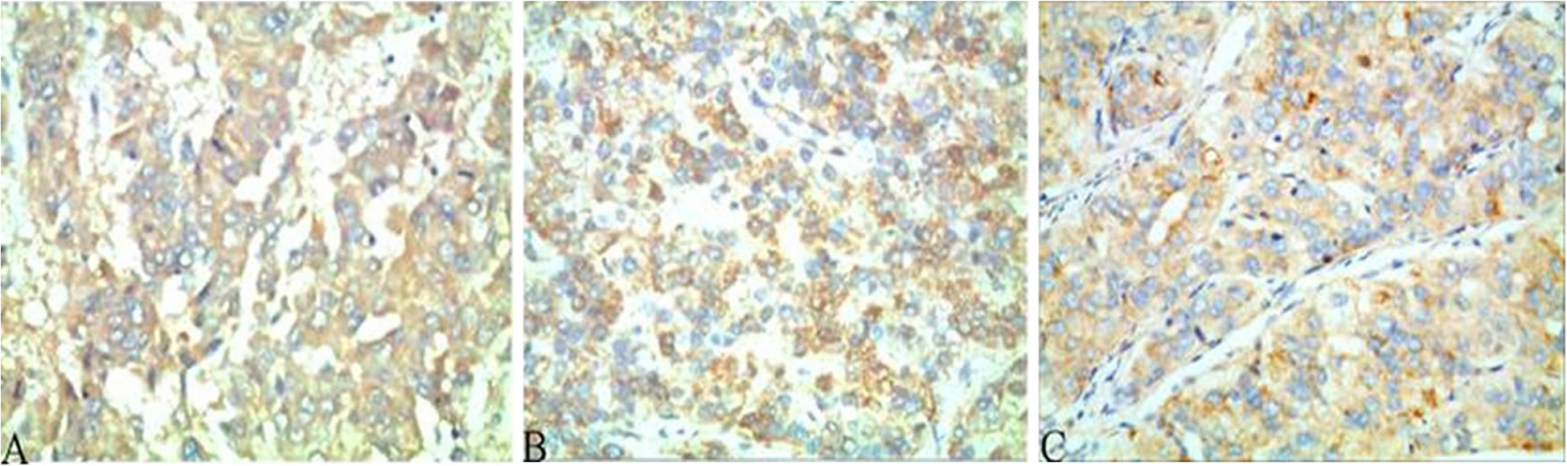
**The expression of Bcl-2 (A), Bax (B) and Survivin (C) in HCC.** All three were expressed in the cytoplasm. Magnification × 400.

**Figure 6:**
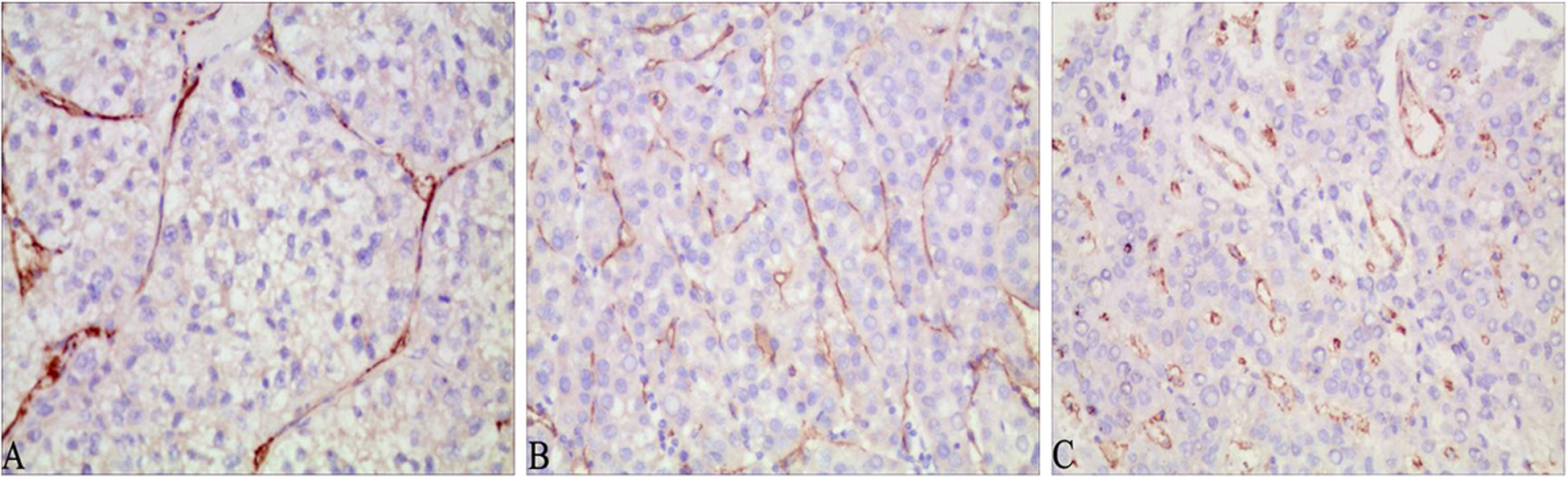
**CD34 expression with lower (A), middle (B) and higher (C) MVD in HCC.** Magnification × 200.

Nuclear and cytoplasmic expression of the proliferation-related proteins PCNA and NET-1, respectively, were detected by immunohistochemical staining in HCC (Figure 4). The Bcl-2, Bax and Survivin apoptosis-related proteins showed cytoplasmic staining (Figure 5). CD34 expression in stromal microvessels exhibited a scattered pattern (Figure 6). In the 103 HCC cases, the average MVD was 198 ± 96/0.64 mm^2^. The cases were divided into three grades: higher MVD (34 cases) with an average of 308 ± 46/0.64 mm^2^, middle MVD (35 cases) at 194 ± 23/0.64 mm^2^ and lower MVD (34 cases) at 91 ± 37/0.64 mm^2^.

**Figure 7:**
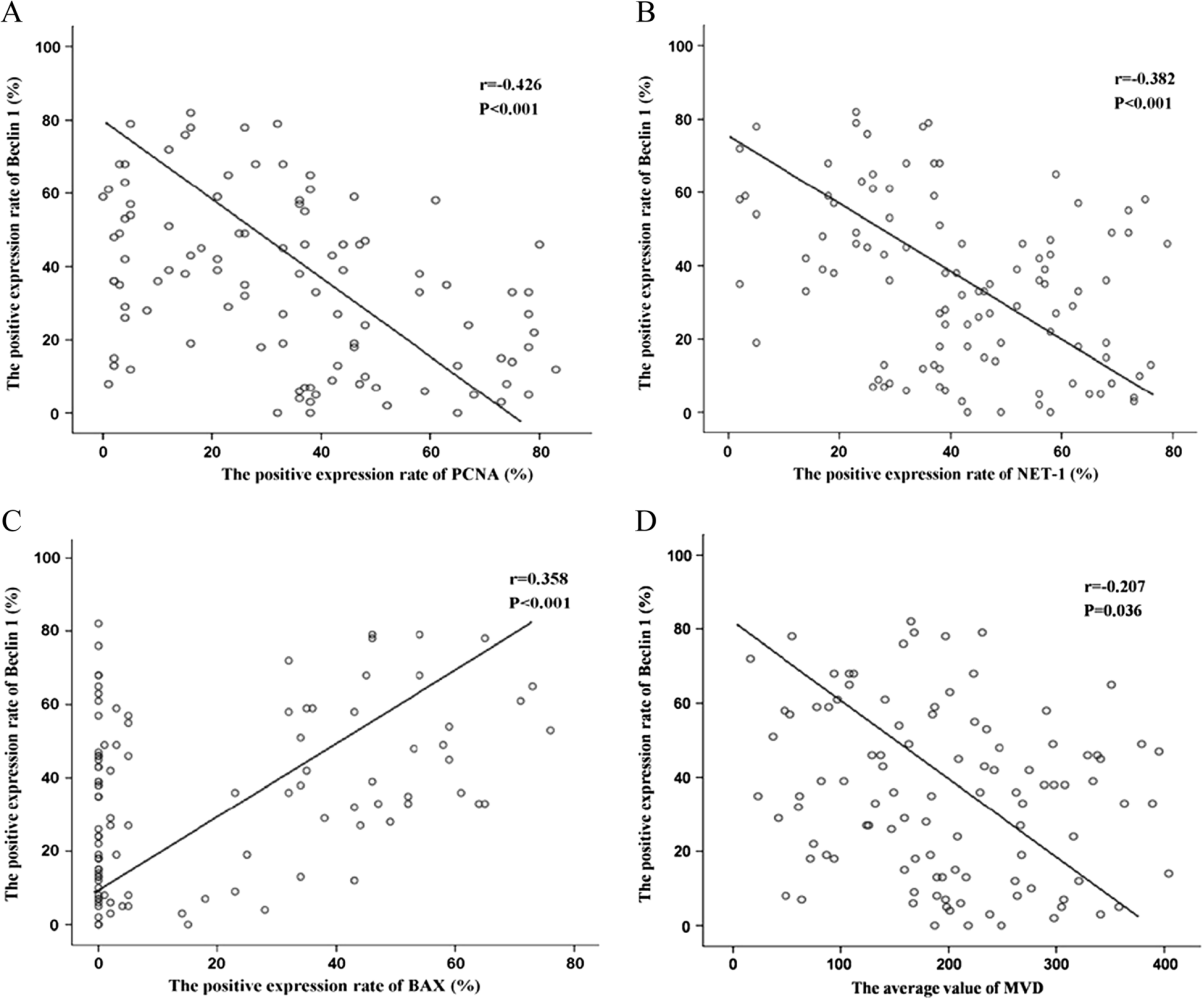
**The relationship between Beclin-1 and PCNA (A), NET-1 (B), Bax (C) and MVD (D) by Pearson related analysis.** The scatter plot graph showed that Beclin-1 was negatively related to PCNA, NET-1, and MVD, and positively related to Bax.

As seen in Table 3, Spearman related analysis shows the association between the expression of Beclin-1 and proliferation and apoptosis related proteins and MVD in the 103 HCC cases. Significant negative correlations between the expression of Beclin-1 and PCNA (r = −0.245,*P* = 0.013), Beclin-1 and NET-1 (r = −0.382. *P* < 0.001), Beclin-1 and Bcl-2 (r = −0.226, *P* = 0.021), and Beclin-1 and MVD (r = −0.292, *P* = 0.003) were observed. Conversely, a significant positive relationship between the expression of Beclin-1 and Bax (r = 0.242, *P* = 0.014) was identified. However, no significant relationship between the expression of Beclin-1 and Survivin (r = 0.044, *P* = 0.659) was seen. Furthermore, Pearson related analysis demonstrated that Beclin-1 was negatively related to PCNA (r = −0.426, *P* < 0.001), NET-1 (r = −0.382, *P* < 0.001), and MVD (r = −0.207, *P* < 0.01), whereas it was positively related to Bax (r = 0.358, *P* < 0.001) (Figure 7). Although Beclin-1 was negatively related to Bcl-2 according to Spearman related analysis, no significant relationship between the two was identified by Pearson related analysis (*P* > 0.05).

**Table 3:** The correlation of the expression of Beclin-1 with either proliferation and apoptosis related proteins or MVD in HCC

**Related proteins**	**N**	**Beclin-1 expression**						**r/P**
		**(—)**	**%**	**(+)**	**%**	**(++)**	**%**	
PCNA								−0.242/0.014*
-	21	4	19.0	8	38.1	9	42.9	
+	62	16	25.8	35	56.5	11	17.7	
++	20	8	40.0	10	50.0	2	10.0	
NET-1								−0.245/0.013*
-	7	0	0.0	2	28.6	5	71.4	
+	59	15	25.4	32	54.2	12	20.3	
++	37	13	35.1	19	51.4	5	13.5	
Bcl-2								−0.226 /0.021*
-	73	16	21.9	38	52.1	19	26.0	
+	19	7	36.8	10	52.6	2	10.5	
++	11	5	45.5	5	45.5	1	9.1	
Bax								0.242/0.014*
-	51	21	41.2	21	41.2	9	17.6	
+	27	5	18.5	14	51.9	8	29.6	
++	15	2	13.3	8	53.3	5	33.3	
Suvivinin								0.044/0.659
-	39	12	30.8	20	51.3	7	17.9	
+	43	10	23.3	22	51.2	11	25.6	
++	21	6	28.6	11	52.4	4	19.0	
MVD								−0.292/0.003*
Higher	34	11	32.4	22	64.7	1	2.9	
Middle	35	15	42.9	8	22.9	12	34.3	
Lower	34	2	5.9	23	67.6	9	26.5	

*p<0.05.

### The association of Beclin-1 expression with survival analysis of HCC

**Figure 8:**
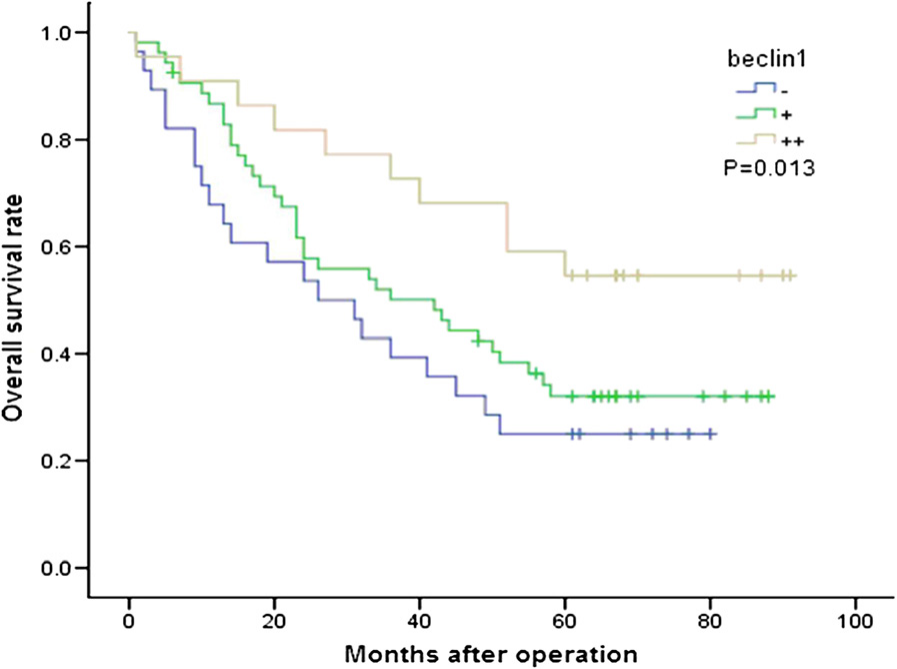
**Kaplan-Meier survival curves stratified for Beclin-1 expression in whole study population.** The median OS of Beclin-1 (++) group was much higher than the other two groups.

The association between Beclin-1 expression and patient survival was investigated using Kaplan-Meier analysis and Log-rank test with single-factor and multivariate analysis for the follow-up data from the 103 HCC cases. The median overall survival (OS) after operation for the 103 patients was 41 months (ranging from 1 to 91 months). Kaplan-Meier analysis of patient survival revealed that low expression of Beclin-1 may point to a poor prognosis for HCC patients. The median OS of the Beclin-1 (++), (+) and (−) groups was 62, 36 and 28.5 months, respectively, with the log-rank test demonstrating significant difference (*P* = 0.013, Figure 8). The prognosis of the Beclin-1 (++) group was better than that of the (+) group (*P* = 0.036) and the (−) group (*P* = 0.008), although no significant difference in OS was observed between Beclin-1 (+) and (−) groups (*P* = 0.281).

**Figure 9:**
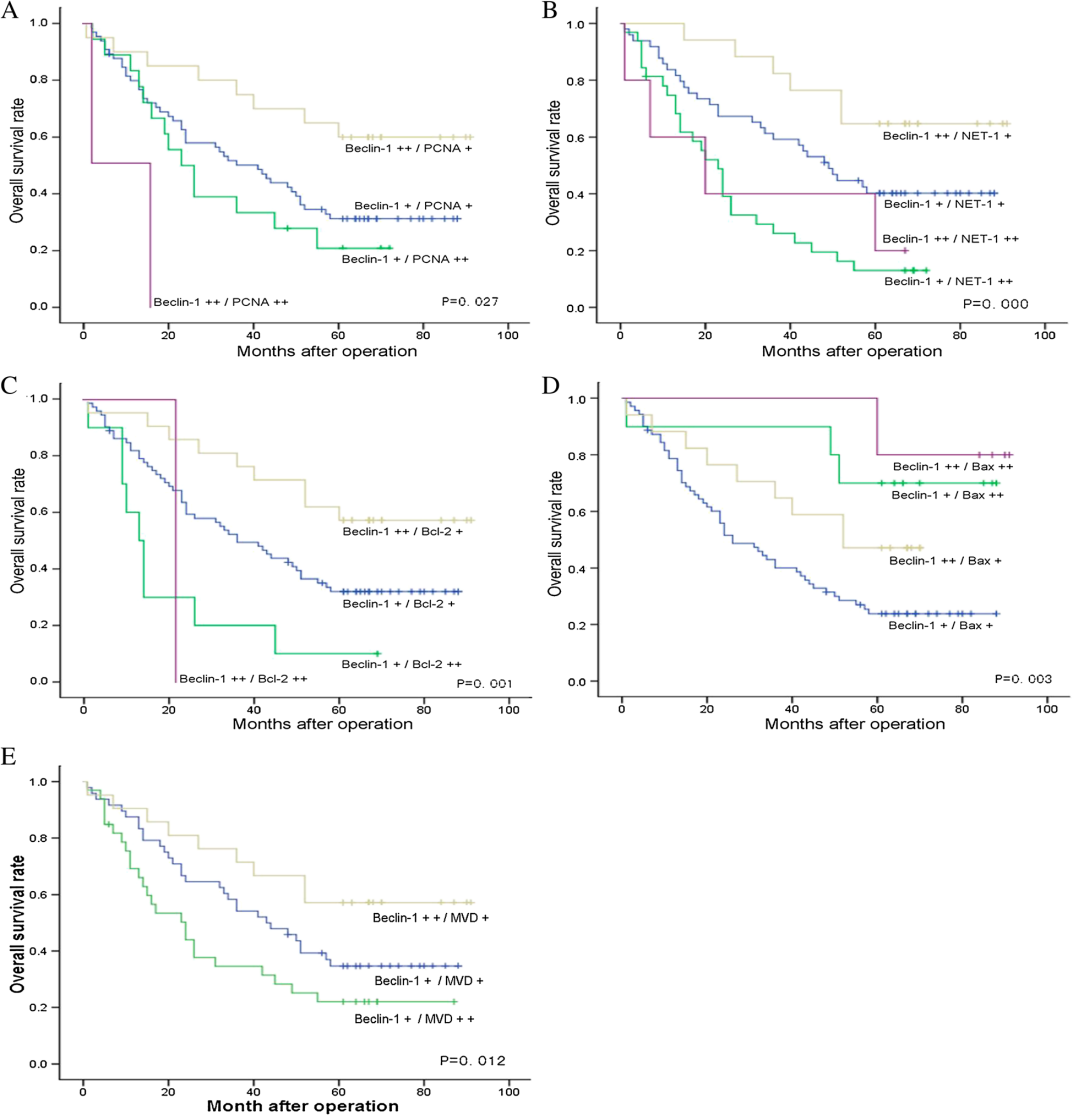
**Kaplan-Meier survival curves of HCC patients with Beclin-1 and PCNA co-expression (A), Beclin-1 and NET-1 co-expression (B), Beclin-1 and Bcl-2 co-expression (C), Beclin-1 and Bax co-expression (D), and Beclin-1 and MVD co-expression (E) (log-rank test, *P*** **< 0.05).**

The associations between Beclin-1 with either PCNA, NET-1, Bcl-2, Bax expressions or CD34 positive MVD as well as patient survival were investigated. All cases were further divided into the following groups according to co-expression of Beclin-1 and each of the four genes and MVD. Because no significant difference in OS was observed between the Beclin-1 (+) group and (−) group, both groups were evaluated together as a low expression group. The other four genes (PCNA, NET-1, Bcl-2 and Bax) and MVD groups, both the middle and lower groups, were also treated the same by combining them into a low expression group and low MVD group (+); whereas the (++) group was an overexpression group and higher MVD group (++). The 5-year OS of cases with positive co-expression of both genes were investigated using Kaplan-Meier analysis (Figure 9) and Log-rank test (Table 4). The results indicated that the survival rates were significantly related to Beclin-1 and PCNA expression (*P* = 0.027), Beclin-1 and NET-1 expression (*P* = 0.000), Beclin-1 and Bcl-2 expression (*P* = 0.001), Beclin-1 and Bax expression (*P* = 0.003), and Beclin-1 and MDV (*P* = 0.003). The 5-year OS of cases with co-expression of Beclin-1 (++)/PCNA (+) was significantly higher than that in cases with Beclin-1 (+)/PCNA (+) and Beclin-1 (+)/PCNA (++) (65.0 vs. 30.2% and 16.7%, respectively; *P* < 0.01). Cases with Beclin-1 (++)/NET-1 (+) showed a significantly higher 5-year OS than cases with Beclin-1 (+)/NET-1 (+) (64.7 vs. 36.7%, respectively; *P* = 0.046); the 5-year OS of cases with Beclin-1 (++)/Bcl-2(+) was significantly higher than that of cases with Beclin-1 (+)/Bcl-2 (++) and Beclin-1 (+)/Bcl-2 (+) (61.90 vs. 10% and 29.6%, respectively; *P* < 0.01), and 5-year OS in cases with Beclin-1 (++)/Bax (+) and Beclin-1 (+)/Bax (++) were significantly higher than cases with Beclin-1 (+)/Bax (+) (47.1 and 70.0% vs. 21.1%, respectively; *P* < 0.01). The cases with Beclin-1 (++)/MVD-middle showed significantly higher 5-year OS than cases with Beclin-1 (+)/MVD-high (57.1 vs. 21.2%, respectively; *P* < 0.01) and Beclin-1(+)/MVD-middle (57.1 vs. 31.3%, respectively; *P* < 0.05). In addition, 5-year OS of cases with Beclin-1 (+)/Bcl-2 (+) was significantly higher than cases with Beclin-1 (+)/Bcl-2 (++) (29.6 vs. 10%, respectively; *P* = 0.015), and 5-year OS in cases with Beclin-1 (+)/NET-1 (+) was significantly higher than that in cases with Beclin-1 (+)NET-1 (++) (36.7 vs. 12.5%, *P* < 0.001). The 5 year OS was 100%, 100% and 0% in cases with Beclin-1 (++)/Bax (++) (5/5), Beclin-1 (++)/MVD-high (1/1) and Beclin-1 (++)/PCNA (++) (0/2). No other significant statistical differences among the other groups were found (*P* > 0.05).

**Table 4:** The 5-year overall survival rate of patients with both genes co-expression

**Groups**	**Gene expression**	**Cases**	**Survival**	**5-year OS (%)**	**Compared**	**Rank test**
a	Beclin-1++/PCNA++	2	0	0		
b	Beclin-1++/PCNA+	20	13	65.0	b vs. c	P=0.008
c	Beclin-1+/PCNA++	18	3	16.7	b vs. d	P=0.025
d	Beclin-1+/PCNA+	63	19	30.2	C vs. d	P>0.05
e	Beclin-1++/NET-1++	5	2	40.0	E vs. f	P>0.05
f	Beclin-1++/NET-1+	17	11	64.7	f vs g	P=0.000
g	Beclin-1+/NET-1++	32	4	12.5	h vs g	P=0.003
h	Beclin-1+/NET-1+	49	18	36.7	f vs h	P=0.046
i	Beclin-1++/Bcl-2++	1	0	0		
j	Beclin-1++/Bcl-2+	21	13	61.9	J vs k	P=0.000
k	Beclin-1+/Bcl-2++	10	1	10.0	J vs l	P=0.029
l	Beclin-1+/Bcl-2+	71	21	29.6	l vs k	P=0.015
m	Beclin-1++/Bax++	5	5	100.0		
n	Beclin-1++/Bax+	17	8	47.1	n vs. o	P>0.05
o	Beclin-1+/Bax++	10	7	70.0	n vs. p	P>0.05
p	Beclin-1+/Bax+	71	15	21.1	o vs. p	P=0.010
q	Beclin-1++/MVD++	1	1	100.0		
r	Beclin-1++/MVD+	21	12	57.1	r vs. s	P=0.000
s	Beclin-1+/ MVD++	33	3	21.2	r vs. t	P=0.043
t	Beclin-1+/ MVD+	48	15	31.3	s vs. t	P>0.05

+, including – and +.

Cox regression analysis was used to compare Beclin-1 expression with clinicopathological features of survival prediction (Table 5). As shown in Table 5, using univariate Cox regression analysis, the parameters such as tumor size (>5 cm), Edmondson grades (III + IV), TNM stage (III + IV), vascular invasion and capsular infiltration and Beclin-1 expression (− and +) were significantly associated with poor OS. By multivariate Cox regression analysis, only large tumor size, higher Edmondson grades, late stage and loss of or lower expression of Beclin-1 were identified to be independent prognostic factors for OS (*P* < 0.05). Together this showed that Beclin-1 together with tumor size, tumor differentiation, and TNM stages were strongly associated with OS. However, other variables including age, sex, cirrhosis background, capsular infiltration, serum AFP level, HBsAg status and multinodular tumor seemed to show a general trend, with subtle differences in survival without significance.

**Table 5:** Univariate and multivariate Cox regression analysis for overall survival based on Beclin-1 expression and clinicopathological variables

**Variables**	**Univariate analysis**		**Multivariate analysis**	
	**Hazard ratio (95% CI)**	**P**	**Adjusted hazard ratio (95% CI)**	**P**
Age	0.814 (0.500-1.325)	0.407		
Sex	1.139 (0.621-2.091)	0.673		
Tumor size ( ≤ 5 cm vs. >5 cm)	2.408 (1.474-3.935)	0.000	2.532 (1.539-4.165)	0.000
Edmondson grades (I+II vs. III+IV)	1.421 (1.109-1.821)	0.005	1.365 (1.062-1.753)	0.015
TNM stage (I+II vs. III+IV)	1.566 (1.217-2.014)	0.000	1.948 (1.410-2.692)	0.000
Capsular infiltration (Negative vs. Positive)	0.555 (0.340-0.906)	0.018		0.896
Vascular invasion (Negative vs. Positive)	1.911 (1.162-3.143)	0.011		0.999
Cirrhosis background	1.586 (0.809-3.110)	0.179		
Serum AFP level	1.606 (0.961-2.683)	0.071		
HbsAg status	1.317 (0.689-2.516)	0.405		
Multinodular tumor (Single vs. Multiple)	0.998 (0.521-1.909)	0.995		
Beclin-1 [(−+) vs. (++)]	0.690 (0.492-0.967)	0.031	0.667 (0.473-0.941)	0.021

## Discussion

Autophagy is a tightly-regulated catabolic process that involves the degradation of intracellular components via lysosomes during cell growth, development and tumorigenesis [25]. The role that autophagy plays in oncogenesis is double sided and dependent on context. In a tumor microenvironment, autophagy can serve as a means of temporary survival in response to metabolic stress. However, as cellular stress continues to result in continuous or progressive autophagy, cell death would follow. Moreover, emerging evidence has revealed autophagic features in cells treated with chemotherapeutic agents. Whether the high level of autophagy induced by cytotoxic drugs should be regarded as a direct cell death execution pathway or a garbage disposal mechanism whereby cells preserve their viability for long-time survival is still not clear [26]. Currently, numerous studies have examined the molecular mechanism of autophagy to elucidate the role that autophagy plays in cancer initiation and progression. Beclin-1, the mammalian ortholog of Atg6, is required for a proximal step in autophagy [27], recruiting proteins from the cytoplasm for autophagic degradation or in supplying the autophagic pathway with membrane components [18]. Zou M et al. found that Oroxylin A, a natural mono-flavonoid extracted from Scutellariae radix, induced Beclin-1-mediated autophagy in human HCC HepG2 cells [28]. Inactivation of Beclin-1 has been demonstrated to result in increased tumorigenesis in mice. Beclin-1 is monoallelically deleted in a high percentage of human breast, ovarian and prostate cancers, and decreased levels of the protein have been found in human breast, ovarian and brain tumors [24,29-31]. Moreover, enforced expression of Beclin-1 has been shown to inhibit the formation of human breast tumors in mouse models [24,32]. Therefore, Beclin-1 could play a role as a tumor suppressor and its decreased expression may contribute to the development of human cancer.

In our study, Beclin-1 was mainly located in the cytoplasm of HCC tissues, along with a significantly decreased level of expression in HCC tissues compared with the adjacent nontumor tissues. These results are similar to those by Liang et al., in which lower expression of Beclin-1 was found in human breast epithelial carcinoma cell lines and tissues, and high levels of Beclin-1 expression was expressed ubiquitously in normal breast epithelial as detected by western blot and immunohistochemistry [30]. Another study showed that transfection of Beclin-1 into a transformed breast carcinoma cell line decreased its tumorigenic potential in nude mice [27]. These findings suggested that these carcinomas might possess defective autophagy. Other findings revealed that autophagy enhanced tumor development and protected tumor cells from stimuli that result in cell death [11,33]. Ahn et al. reported that the increased Beclin-1 expression in colorectal and gastric cancer cells compared with normal epithelial cells suggests that neo-expression of Beclin-1 may play a role in both colorectal and gastric tumorigenesis [34]. In *in vivo* HCC xenograft tumors, induced autophagy led to inhibition of tumor growth [28], which supports an autophagy-mediated antitumor activity. These divergent results also suggest that Beclin-1 may function in a tissue-specific manner.

In this study, expression level of Beclin-1 was negatively correlated with HCC Edmondson grades, HCC with cirrhosis background and vascular invasion. We found that Beclin-1 expression was higher in HCC with Edmondson I–II grade than that with III–IV grade. Similarly, Beclin-1 expression was significantly lower in HCC with vascular invasion and cirrhosis background than HCC without vascular invasion and cirrhosis. These results indicate that Beclin-1 may be an immediate-early response gene in tumorigenesis. The absence of Beclin-1 could be an early event in the process of HCC, and thus play a more critical role in HCC progression.

To examine the underlying mechanisms of how Beclin-1 affects HCC malignant transformation, the association of Beclin-1 expression in HCC with cellular proliferation-related proteins such as PCNA and NET-1, and apoptosis-related proteins including Bcl-2, Bax and Survivin were studied. PCNA antigen is a nuclear antigen expressed in proliferating cells in all stages of the cell cycle except stage G0 and serves as a marker for proliferation. NET-1, a member of the transmembrane 4 superfamily (TM4SF), acts as a molecular service protein that enhances the formation and stability of functional signal transduction complexes by connecting specific cell surface proteins, such as lineage specific proteins, integration proteins and other TM4SFs members. NET-1 thus plays an important role in cell signal transduction, regulation, adhesion, migration, proliferation and differentiation [5,6]. Previous studies have shown that NET-1 expression was related closely to HCC proliferation, with significant upregulation during formation of cancers [14]. Bcl-2 is one of the main anti-apoptotic proteins normally residing in the mitochondrial membrane and cytoplasm. When cells are deprived of survival signals or subjected to stress, Bcl-2 is released from the mitochondrial membrane and replaced by pro-apoptotic factors, such as Bax. When Bcl-2 levels decrease, the permeability of the mitochondrial membrane increases, and several proteins that can activate the caspase cascade are released. In this present study, Spearman related analysis showed a significant negative relationship between Beclin-1 and PCNA, between Beclin-1 and NET-1, and between Beclin-1 and Bcl-2. A significant positive relationship was observed between Beclin-1 and Bax. However, no significant relationship was observed between Beclin-1 and Survinin. Pearson related analysis demonstrated that Beclin-1 was negatively related to PCNA and NET-1, and positively related to Bax, but no significant relationship was detected between Beclin-1 and Bcl-2, which might be owed to the fact that the number of Bcl-2 positive cases was too low to be representative in the total cases. Bcl-2 is an interacting partner of Beclin-1 and negatively regulates its autophagy function [35]. In the present work, we did not assay the expression of autophagy markers such as LC3 or p62/SQSTSM1 [36]. With relevance to our findings, a previous study showed that high expression of Beclin-1 correlated with a good prognosis in non-Hodgkin lymphoma when the tumors were Bcl-2 low or negative expressing and positive for the autophagy marker LC3 [36]. Because Bax is a pro-apoptosis factor and Bcl-2 is an apoptosis inhibitor, our data identifying an association of Beclin-1 expression with Bax overexpression suggests that cellular autophagy may be positively related to apoptosis in HCC. Similarly, Zou M et al. also observed that autophagy occurred prior to noticeable apoptosis in HepG2 cells. We propose a novel function for autophagy in promoting death of HCC cells, which involves inhibiting cell proliferation and promoting cell apoptosis. However, the mechanisms underlying this novel function remain unclear.

Angiogenesis plays an important role in the malignant transformation, growth, and metastasis of parenchymal tumors. Tumor angiogenesis is regulated by angiogenesis factors generated and excreted by tumor cells. HCC is a highly vascularized tumor that requires the formation of numerous blood vessels to receive sufficient blood supply to grow and proliferate. Consequently, angiogenesis is a crucial process in the development of HCC. In our HCC group, Spearman related analysis and Pearson related analysis demonstrated that Beclin-1 expression negatively correlated to MVD.

Cellular proliferation, apoptosis and angiogenesis are important in cancer occurrence, development and progression processes. As tumor associated factors, the expressions of PCNA and NET-1 are closely related to cell proliferation. The elevated expression of Bax and decreased expression of Bcl-2 leads cells to activate apoptosis to higher levels. Increasing Beclin-1 expression in HCC cells was always accompanied by lower MVD. Therefore, we hypothesized that the co-expression of these genes in HCC has a synergistic effect through inhibiting proliferation and promoting both apoptosis and angiogenesis, which greatly reduces the malignant progression of cancers. The prognostic significance of Beclin-1 has been studied in several types of solid tumors, and decreased expression of Beclin-1 was correlated with tumor progression in breast, ovarian and brain cancers [21-24]. In our study, Kaplan-Meier analysis showed that the patients with higher expression of Beclin-1 had longer OS, suggesting that Beclin-1 expression could indicate prognosis of HCC. Our findings are consistent with those previously observed, showing that decreased Beclin-1 expression was correlated with a lower survival rate in patients with esophageal cancer, glioblastoma and colon cancers [22,24,37], and with the observation that high expression of Beclin-1 correlates with a better survival rate in non-Hodgkin lymphoma patients [36].

Several authors have reported the expression and significance of these genes (Beclin-1, PCNA, NET-1 and Bcl-2) in HCC [8,16,17], but until now, no study has examined the correlations among expression of these genes in HCC and the relationship between co-expression of these genes and survival of HCC patients. This study focused on significance of the co-expression of related genes in HCC prognosis. In the present study, co-expression patterns such as low Beclin-1 with high PCNA expression, low Beclin-1 with high NET-1 expression, and low Beclin-1 with high Bcl-2 expression were associated with an increased risk of HCC progression. The co-expression was also associated with malignant progression and poor prognosis of patients. In addition, the 5-year OS of patients with co-expression of higher Beclin-1 expression with the lower expression of PCNA, NET-1, Bcl-2 or lower MVD was significantly higher than other expression patterns. This is the first report showing that co-expression of Beclin-1 and PCNA, NET-1, Bcl-2, or Bax, or MVD is associated with HCC patient prognosis. The observations also indirectly support the concept that synergistic effects of Beclin-1 and Bax may contribute to better prognosis by simultaneously promoting autophagy and apoptosis in HCC.

Some of the clinicopathological factors and the expression of Beclin-1 selected by univariate Cox regression analysis showed significant difference in HCC survival. Tumor size (>5 cm), Edmondson grades (III + IV), TNM stage (III + IV), vascular invasion, capsular infiltration and low expression of Beclin-1 (− and +) were significantly associated with poor OS. In addition, multivariate Cox regression analysis showed that only large tumor size (>5 cm), higher Edmondson grades (III + IV), late stage (TNM stage III + IV) and loss or lower expression of Beclin-1 were independent prognostic factors for OS Beclin-1, together with tumor size, tumor differentiation, and TNM stages, were strongly associated with OS. The mechanisms underlying the involvement of these factors in HCC need further investigation.

## Conclusion

Our study shows that the expression of the autophagic gene Beclin-1 and its autophagic activities are suppressed in some HCC tissues. Autophagy defects may be associated with tumor progression and poor prognosis of HCC. The expression of Beclin 1 may be a valuable marker to estimate HCC progression. However, further studies are required to confirm these findings and to provide better understanding of the autophagy mechanism in the development of HCC.

## Competing interest

The authors declare that they have no competing interests.

## Authors’ contributions

LC conceived and designed the research; DMQ collected, organized the patients’ information and drafted the manuscript. DMQ, GLW, YYX, SH, XLC and YXZ analyzed data; DMQ interpreted results of experiments; JQ and JMZ prepared figures; LC and DMQ edited and revised the manuscript; DMQ approved the final version of manuscript; DMQ, YYX, JMZ and QE performed experiments. All authors read and approved the final manuscript.

## Pre-publication history

The pre-publication history for this paper can be accessed here:

http://www.biomedcentral.com/1471-2407/14/327/prepub
